# Structural and Biochemical Characterization of the Nucleosome Containing Variants H3.3 and H2A.Z

**DOI:** 10.3390/epigenomes8020021

**Published:** 2024-05-27

**Authors:** Harry Jung, Vladyslava Sokolova, Gahyun Lee, Victoria Rose Stevens, Dongyan Tan

**Affiliations:** 1 Department of Pharmacological Sciences, Stony Brook University, Stony Brook, New York, NY 11794, USA; harry.jung@yale.edu (H.J.); vladyslava.sokolova@stonybrook.edu (V.S.); gahyun.lee@stonybrook.edu (G.L.); vstevens@chembio.com (V.R.S.); 2 Department of Pharmacology, Yale University, New Haven, CT 06520, USA; 3 Chembio Diagnostics Inc., Medford, NY 11763, USA

**Keywords:** histone variant, H3.3, H2A.Z, nucleosome, chromatin

## Abstract

Variant H3.3, along with H2A.Z, is notably enriched at promoter regions and is commonly associated with transcriptional activation. However, the specific molecular mechanisms through which H3.3 influences chromatin dynamics at transcription start sites, and its role in gene regulation, remain elusive. Using a combination of biochemistry and cryo-electron microscopy (cryo-EM), we show that the inclusion of H3.3 alone does not induce discernible changes in nucleosome DNA dynamics. Conversely, the presence of both H3.3 and H2A.Z enhances DNA’s flexibility similarly to H2A.Z alone. Interestingly, our findings suggest that the presence of H3.3 in the H2A.Z nucleosome provides slightly increased protection to DNA at internal sites within the nucleosome. These results imply that while H2A.Z at active promoters promotes the formation of more accessible nucleosomes with increased DNA accessibility to facilitate transcription, the simultaneous presence of H3.3 offers an additional mechanism to fine-tune nucleosome accessibility and the chromatin environment.

## 1. Introduction

The regulation of gene expression in eukaryotes is achieved through alterations in the structure and functions of chromatin. The epigenetic mechanisms exist to regulate this process include histone post-translational modifications (PTMs), ATP-dependent chromatin remodeling, and histone variant exchange. Histone variant exchange, the process of depositing and removing histone variants, is a key epigenetic mechanism that has a functional role in multiple nuclear processes. Unlike their canonical counterparts, which are synthesized and incorporated during interphase [1], histone variants are expressed throughout the cell cycle [2]. The process of depositing and removing histone variants is an energy-consuming process that is facilitated by histone chaperones and/or ATP-dependent chromatin remodelers. Given their important and specialized functions in genome regulation, it is therefore not surprising that variant dysregulation is implicated in a variety of diseases such as tumorigenesis and developmental defects [3].

**Figure 1:**
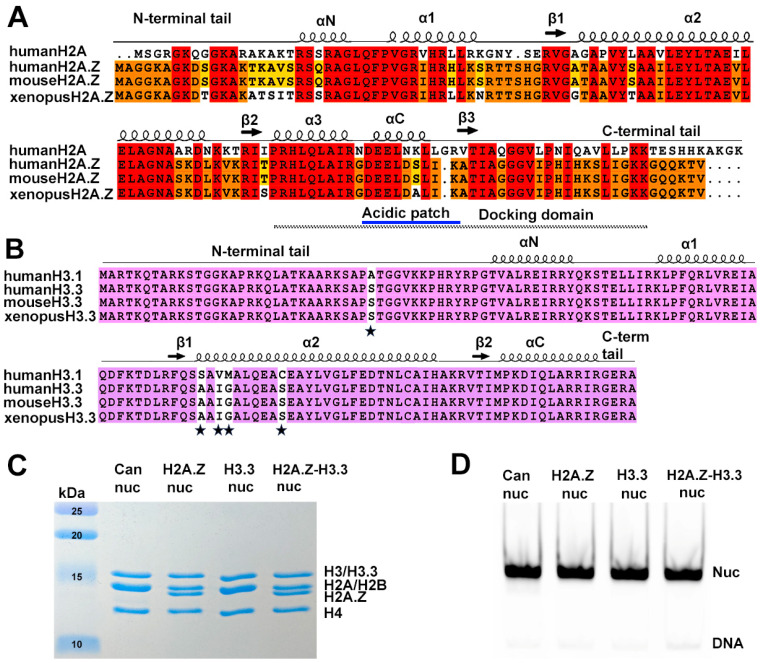
Sequence conservation of histone variants and nucleosome preparation. (**A**) Sequence alignment of variant H2A.Z and canonical H2A, showing identical sequences between mouse and human H2A.Z. Conserved amino acids among the three H2A.Z proteins and H2A are highlighted in red. Residues conserved in H2A.Z across all three species are marked in orange, while those conserved only between human and mouse are shown in yellow. Structural elements are indicated above alignment. Docking domain indicated as a dotted line under the alignment. (**B**) Sequence alignment of the canonical H3.1 and the variant H3.3, showing identical sequences between mouse and human H3.3. Conserved amino acids are in purple. The five amino acid substitutions are marked by stars. (**C**) Purified histone octamers revealed using 15% Coomassie-stained SDS-PAGE gel. (**D**) In vitro reconstituted nucleosomes revealed using 3% Native-PAGE.

Several histone variants are known to be involved in transcriptional control. Among these is variant H2A.Z, an essential protein for the survival of several organisms [4,5,6,7]. Variant H2A.Z shares only ~60% sequence identity with its canonical counterpart H2A, yet it is highly conserved across species (Figure 1A). H2A.Z has been linked to both transcription activation and repression. On one hand, H2A.Z is predominantly located at the distal end of inducible promoters. This localization is crucial for poising the gene for rapid activation [8,9,10,11,12]. Upon transcription activation, H2A.Z is replaced by the canonical histone H2A [13,14,15]. Nevertheless, a substantial amount of H2A.Z is also found at heterochromatin regions such as the centromere and pericentromeric heterochromatin [16,17,18,19,20], as well as in the gene bodies of repressed genes [21,22]. Recent studies, including ours, have revealed that the incorporation of H2A.Z increases the accessibility of entry/exit DNAs on nucleosomes [23,24]. Furthermore, we demonstrate that, in vivo, the presence of H2A.Z enables nucleosome arrays to fold into a more compact and regular higher-order structure [23].

A second histone variant, H3.3, is primarily found in active transcribing genes, promoters, and gene regulatory elements. It is generally considered as an active marker for transcription [25,26,27]. Contrary to H2A.Z, H3.3 is different from the canonical H3 histone only in four or five amino acids (Figure 1B). An early study found that H3.3 nucleosomes are much more susceptible to salt-dependent disassembly than canonical nucleosomes [28]. Nevertheless, later studies showed conflicting results, indicating that H3.3 alone has a negligible effect on nucleosomes’ structure [29] and stability in vivo [30]. Therefore, it remains unclear how H3.3-specific features contribute to distinct properties in H3.3 nucleosomes.

Nucleosomes containing both H2A.Z and H3.3 variants also exist in vivo, being primarily located downstream of the nucleosome-free regions of active promoters [26,28]. The physical properties and stability of these double-variant nucleosomes remain controversial. Initially, double-variant nucleosomes isolated from vertebrates were shown to be unstable and sensitive to salt-dependent disruption, with a tendency to lose H2A.Z/H2B dimers [28]. However, another study found only subtle changes in the stability of these double-variant nucleosomes in vitro [31]. From a structural standpoint, the mechanism by which H3.3-specific residues confer new properties to the nucleosome remains elusive. One early crystallographic study demonstrated minimal changes in the nucleosome structure when both variants are present [32]. Beyond the nucleosome level, variant H3.3 was shown to inhibit H2A.Z-mediated chromatin higher-order structure formation [30]. This raises questions about how variant H3.3 interfaces with H2A.Z to modulate chromatin structure and function at different chromatin regions.

Several recent cryo-EM studies have shed light on variant-specific changes in nucleosomes and chromatin. Despite the different variants involved, a common theme emerges: histone variants exert their influence on chromatin by modulating DNA near the entry/exit sites [33]. To further understand H3.3-mediated chromatin changes and transcriptional regulation, we compared nucleosomes containing canonical histones, variants H3.3, and H2A.Z (in isolation and in combination), using DNA accessibility assays to assess variant-dependent DNA dynamics. Our biochemical data show that H3.3 alone does not alter linker DNA accessibility on nucleosomes. However, the presence of variant H2A.Z, either alone or with H3.3, substantially increases entry/exit DNA flexibility within the nucleosome. Consistent with these biochemical data, our cryo-EM structure of the H2A.Z-H3.3 double-variant nucleosome reveals an overall structure remarkably similar to that of the H2A.Z nucleosome, displaying asymmetric DNA wrapped around the histone octamer. Furthermore, our study revealed that the INO80-dependent chromatin remodeler exhibits similar DNA translocation activity on double-variant nucleosomes compared to H2A.Z nucleosomes. Together, these findings suggest that variant H3.3 alone does not alter the entry/exit DNA accessibility on nucleosomes. The concurrent presence of H3.3 with H2A.Z, on the other hand, resembles the effect of H2A.Z on nucleosome stability and DNA dynamics. Intriguingly, the double-variant nucleosome displays a small but significant reduction in DNA accessibility at internal sites compared to the H2A.Z nucleosome in our assay. We propose that the presence of H2A.Z leads to the formation of more accessible nucleosomes with increased DNA ends to facilitate transcription. The concurrent presence of H3.3 offers an additional mechanism to fine-tune the nucleosome DNA accessibility and thus the chromatin environment at promoters and gene-regulatory elements.

## 2. Material and Methods

### 2.1. Protein Production

Histones H2A, H2B and H4, and H3 from *Xenopus laevis* were expressed in BL21 (DE3) pLysS *E. coli* cells. Proteins were purified according to the established procedures [34]. The mouse H3.3 gene was re-cloned into a pET-LIC expression vector containing mCerulean-H3.3-N-14, which was a generous gift from Michael Davidson (Addgene plasmid # 55377; http://n2t.net/addgene:55377 (accessed on 7 May 2024); RRID: Addgene_55377). The mouse H2A.Z.1 gene in a pIND-EGFP vector was a generous gift from Danny Rangasamy (Addgene plasmid # 15770; http://n2t.net/addgene:15770 (accessed on 7 May 2024); RRID:Addgene_15770). The gene was re-cloned into a pET-LIC expression vector. Expression and purification of the histone variants H3.3 and H2A.Z were performed following the same procedure.

All histone octamers used in this study were produced in vitro through refolding, as previously described [34]. Briefly, all histones were mixed in equal molar concentrations, followed by incubation for 2 h in unfolding buffer (7 M guanidine HCl, 20 mM Tris, pH 7.5, and 10 mM DTT) and dialysis against at least three changes of refolding buffer (10 mM Tris, pH 7.5, 1 mM EDTA, 2 M NaCl, and 1 mM DTT) at 4 °C. The octamers were further purified with gel filtration chromatography using a Superdex200 (Cytiva, Wilmington, DE, USA) increase 10/300 GL column.

### 2.2. DNAs

We used a plasmid containing twelve tandem repeats of the 167 bp A601 Widom sequence; 167 bp nucleosomal DNA was liberated via EcoRV restriction enzyme digestion. Subsequently, DNA fragments were further purified with anion exchange chromatography using an HQ Poros column (Applied Biosciences). The sequence used for reconstituting the nucleosomes is listed below, with the 601 sequence underlined: ATCCCGCCCTGGAGAATCCCGGTGCCGAGGCCGCTCAATTGGTCGTAGACAGCTCTAGCACCGCTTAAACGCACGTACGCGCTGTCCCCCGCGTTTTAACCGCCAAGGGGATTACTCCCTAGTCTCCAGGCACGTGTCAGATATATACATCCTGTGCATGACTAGAT.

A plasmid with twelve tandem repeats of the 208 bp 601 Widom sequence was also used. This was a generous gift from Dr. Ed Luk. The restriction enzyme ScaI was used to liberate a single repeat of the 208 bp segment. Anion exchange chromatography using an HQ Poros column (Applied Biosystems, Waltham, MA, USA) was used to further purify the DNA fragments. The sequence is as follows, with the 601 sequence: ACTTATGTGATGGACCCTATACGCGGCCGCCCTGGAGAATCCCGGTGCCGAGGCCGCTCAATTGGTCGTAGACAGCTCTAGCACCGCTTAAACGCACGTACGCGCTGTCCCCCGCGTTTTAACCGCCAAGGGGATTACTCCCTAGTCTCCAGGCACGTGTCAGATATATACATCCTGTGCATGTATTGAACAGCGACCTTGCCGGAGT.

End-positioned 0N80 (80 base pairs of extra-nucleosomal DNA at one entry/exit site) Widom DNA was amplified via PCR using the primer pair (0N80-F 5′-CTGGAGAATCCCGGTGCCGAG-3′ and 0N80-R 5′-TCGGTACCCGGGGATCCTCTA-3′) and the plasmid pGEM-3z/601. The latter was a generous gift from Jonathan Widom (Addgene plasmid #26656). The sequence is as follows, with the 601 Widom sequence: CTGGAGAATCCCGGTGCCGAGGCCGCTCAATTGGTCGTAGCAAGCTCTAGCACCGCTTAAACGCACGTACGCGCTGTCCCCCGCGTTTTAACCGCCAAGGGGATTACTCCCTAGTCTCCAGGCACGTGTCAGATATATACATCCTGTGCATGTATTGAACAGCGACCTTGCCGGTGCCAGTCGGATAGTGTTCCGAGCTCCCACTCTAGAGGATCCCCGGGTACCGA.

### 2.3. Nucleosome Reconstitution

Nucleosome reconstitution was carried out by mixing the octamer with the 601 Widom DNA at an equal molar ratio in high-salinity buffer [10 mM Tris, pH 8.0, 2 mM EDTA, 2 M NaCl, and 2 mM 2-Mercaptoethanol (βME)]. The mixture then underwent overnight dialysis in low-salinity buffer (10 mM Tris, pH 8.0, 2 mM EDTA, 5 mM NaCl, and 2 mM βME), as described in [34]. For the HinfI endonuclease digestion assay, nucleosomes containing 208 bp 601 DNA were used. To produce nucleosomes for the Cryo-EM experiments and the MNase digestion assay, 167 bp 601 DNA was used. To produce nucleosomes for the ATP-dependent nucleosome-sliding assay, the end-position 0N80 DNA was used.

### 2.4. HinfI Endonuclease Accessibility Assay

The reactions contained 250 nM of the 208 bp nucleosome and 45 U of the endonuclease enzyme HinfI in Cutsmart buffer (NEB) (20 mM Tris-Ac, pH 7.9, 50 mM KAc, 10 mM MgAc, 100 µg/mL BSA). The total volume of each reaction was 45 µL. The reactions were then incubated at 37 °C. Samples were collected every 15 min (5 µL) and the reaction was quenched by adding 8 µL of a stop buffer (10 mM Tris–HCl, pH 8.0, 0.6% SDS, 40 mM EDTA, 0.1 mg/mL proteinase K). Samples were then incubated at 50 °C for 1 h for deproteination, followed by separation on 8% Native-PAGE gels. The gels were stained with SYBR GOLD and imaged on a Typhoon imager (Cytiva, Wilmington, DE, USA). A quantitative analysis was conducted using ImageJ software version 1.53e. The level of significant difference was determined using a two-way ANOVA test, with *p* ≤ 0.05 being considered significant. The graphical representations and two-way ANOVA tests were completed using Prism 5 software.

### 2.5. MNase Accessibility Assay

In the MNase accessibility assay, 425 nM of the 167 bp nucleosomes were subjected to digestion with 0.75 U of MNase (Roche, Basel, Switzerland) in buffer (10 mM Tris pH 7.4, 50 mM NaCl, 2 mM CaCl_2_) at 37 °C. The total volume of this reaction was 65 µL. Samples (4.5 μL) were collected every 3 min and the reaction was quenched by adding 10 μL of the stop buffer (10 mM Tris pH 7.5, 40 mM EDTA, 0.6% SDS, 0.1 mg/mL proteinase K). The mixture was then incubated at 50 °C for 1 h. The samples were resolved on Native-PAGE gel (19:1 acrylamide/Bis, 2.5% stacking gel with 8% resolving gel) at 4 °C (100 V, 180 min, 1× TBE), followed by staining with SYBR-GOLD (GoldBio, St Louis, MO, USA). The gels were imaged using a Typhoon imager (Cytiva, Wilmington, DE, USA). A quantitative analysis of the gels was performed using ImageJ software. The intensities of the DNA fragments were estimated cumulatively from bands with similar sizes. The percentage change in DNA fragments over time was plotted. The two-way ANOVA test was employed to determine the statistical significance between datasets using the criterion *p* ≤ 0.05. The statistical analyses and graphical representations were completed using Prism 5 software.

### 2.6. Nucleosome-Sliding Assay

A 200 nM amount of end-positioned nucleosomes (0N80) was mixed with INO80-C complexes (50 nM) in sliding buffer (25 mM HEPES pH 8.0, 50 mM NaCl, 5% glycerol, 1 mM TCEP, and 2 mM MgCl_2_) in a final volume of 10 µL at 24 °C. The reaction was incubated at 37 °C for 10 min. Sliding was initiated by adding 1 mM ATP. The reaction was then quenched by adding 5 mM EDTA and 0.2 mg/mL lambda DNA (NEB). Samples were collected at different time points and resolved on 6% Native-PAGE gels in 1× TBE buffer at 4 °C (100 V, 120 min). The gels were stained with SYBR-GOLD (GoldBio) before imaging on a Typhoon imager (Cytiva, Wilmington, DE, USA).

### 2.7. Vitrification

The reconstituted double-variant nucleosomes were concentrated to a final concentration of 6 µM. The samples were cross-linked on ice with 0.1% glutaraldehyde for 15 min. The cross-linking was quenched by adding Tris (pH 8.0) to a final concentration of 50 mM. Aliquots of 3.5 µL of the sample were applied to glow-discharged QUANTIFOIL UltraAuFoil grids with 100 Holey Gold support (R1.2/1.3—300 mesh). Vitrification was performed using a Vitrobot Mark IV (Thermo Fisher, Waltham, MA, USA) at 8 °C under 100% humidity, with a blot time of 4–5 s. Grids were stored in liquid nitrogen until they were imaged.

### 2.8. Cryo-EM Data Collection

Grid screening was conducted using the Talos Arctica microscope (Thermo Fisher, Waltham, MA, USA) at the cryo-EM facility at Stony Brook University. Two of the best grids were selected for data collection, which took place at the UVA Molecular Electron Microscopy Core with the Titan Krios Microscope (Thermo Fisher, Waltham, MA, USA) operating at 300 kV with a Bioquantum energy filter set to zero loss frequency (10 eV). Movies were recorded using a K3 direct electron detector (Gatan Company, Pleasanton, CA, USA) in counting mode with EPU software (version: 2.5.0.4799REL) at a magnification of 81,000×, resulting in a pixel size of 1.08 Å at the specimen level. Defocus values ranged from 1.0 to 2.25 µm. Each movie was dose-fractionated into 40 frames with a dose rate of approximately 1.25 e/pixel/sec. The total exposure time was 2.5 s, corresponding to a total dose of 50 e/Å^2^ per micrograph (Supplementary Materials Table S1). A total of 5140 movie were collected with the two grids.

### 2.9. Image Processing

From the initial dataset, 2673 movies were selected for further data processing after a thorough inspection to remove any suboptimal movies. Movie frames were aligned and summed using MotionCor2 software with patch motion correction [35]. The CTF parameters were estimated using CTFFIND4 [36]. Reference-based auto particle picking was carried out in RELION [37], resulting in a dataset of ~1.2 million particles. Poor-quality particles were removed through 2D classification. Particles with good class averages were pooled and subjected to 3D classification. The best class, containing 205,792 particles, was re-extracted without binning and re-centered, then subjected to consensus 3D refinement. Postprocessing, CTF refinement, and Bayesian Polishing were performed using this consensus-refined map. No symmetry was applied during refinement. These procedures yielded a final map with an average resolution of 3.0 Å.

### 2.10. Model Building and Refinement

The histone core from the H2A.Z nucleosome containing H3.3 (PDB: 5B33) and the 601 DNA sequence from the canonical nucleosome (PDB: 6FQ5) were combined to generate the initial model used for model refinement. This initial model was manually fitted into the density map using UCSF ChimeraX [38], followed by manual rebuilding using Coot [39]. The model was further refined using Phenix.real_space_refine [40]. The model geometry was checked and further idealized according to standard geometry restraints through geometry minimization in Phenix. The statistics are presented in Supplementary Materials Table S1. UCSF ChimeraX was used to visualized the density map and models, as well as for preparing figures for publication.

### 2.11. Quantification and Statistical Analyses

**Figure 2:**
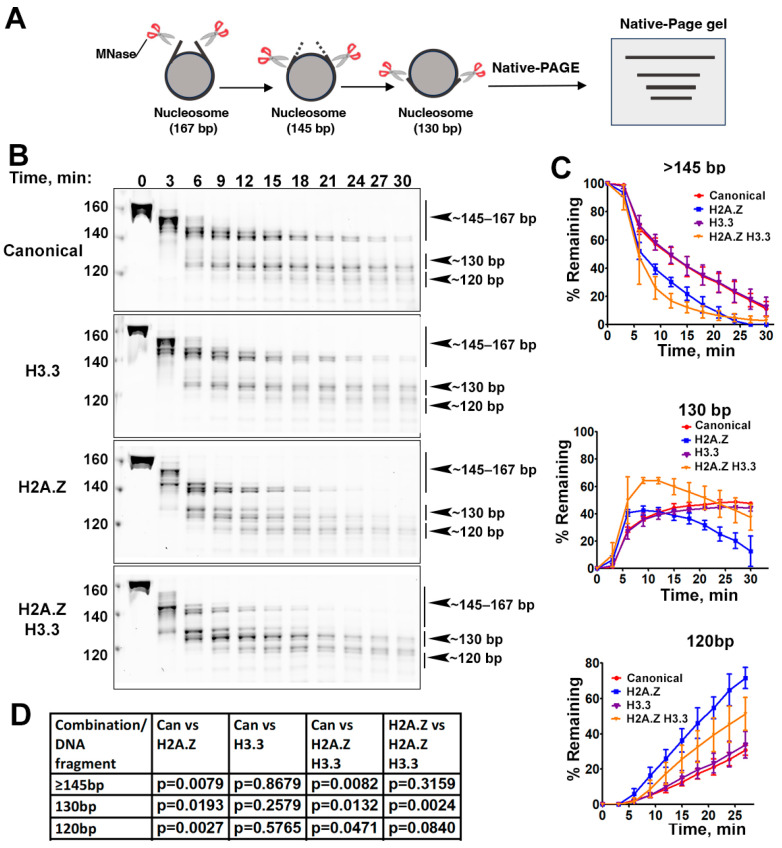
The effects of histone variants on nucleosome DNA accessibility assessed with MNase assays. (**A**) Schematic of the MNase digestion of nucleosomes. (**B**) Representative acrylamide gels of the MNase digestion of canonical, H3.3, H2A.Z, and H2A.Z-H3.3 double-variant nucleosomes, respectively. The digestion products of different sizes (145–167, 130, 120) are labeled. (**C**) Quantification of the digestion DNA products shown in (**B**), representing the fraction of cleaved nucleosome DNA as a function of time. Data are presented as the mean ± SD, *n* = 3. (**D**) *p*-values of the abovementioned quantification analysis, estimated using the two-way ANOVA.

For the experiments depicted in Figure 2 and Figure S1, the average values of three independent experiments were presented alongside their respective standard deviations (SDs). In both instances, consistent and reproducible results were achieved.

## 3. Results

### 3.1. The Incorporation of Double Variant H2A.Z-H3.3 Enhances the Terminal DNA Accessibility on Nucleosomes

To reconstitute nucleosomes for structural and biochemical analyses, we used a DNA fragment containing the Widom 601 nucleosome-positioning sequence [41]. Mono-nucleosomes were reconstituted following an standard protocol [34] using recombinant proteins containing either canonical *Xenopus* histones, mouse histone variant H2A.Z.1, or human histone variant H3.3 (Figure 1C,D).

Previous research has shown that incorporating H2A.Z increases the flexibility and accessibility of terminal DNAs compared to canonical nucleosomes, supported by both restriction enzyme-based assays and cryo-EM studies [23]. While it has been observed that H3.3 nucleosomes are more unstable and susceptible to salt disruption in vitro [28], their specific impact on entry/exit DNA dynamics remains unclear. To address this, we employed a Micrococcal nuclease (MNase)-based DNA accessibility assay to determine the influence of variant H3.3 and its combined effect with H2A.Z on nucleosome DNA accessibility. MNase, a sequence-independent endonuclease, preferentially digests accessible DNA ends on nucleosomes (Figure 2A). The results revealed three dominant DNA fragments (145, 130, and 120 bp) throughout digestion (Figure 2B). We analyzed and quantified the patterns of these fragments across various nucleosome substrates over time to assess the level of compaction or relative DNA accessibility on nucleosomes.

Our findings show that nucleosomes containing the H3.3 variant exhibit DNA protection nearly identical to that of canonical nucleosomes (Figure 2C), indicating that H3.3 alone does not induce detectable structural changes on the entry/exit DNA. In contrast, cleavage of terminal DNAs on the H2A.Z nucleosomes, regardless of the H3.3 variant’s presence, occurred significantly faster than it did in canonical nucleosomes, as evidenced by the rapid disappearance of the 145 bp fragment (top graph in Figure 2C). This aligns with the notion that H2A.Z incorporation enhances DNA breathing at the entry/exit sites [23,24]. Intriguingly, digestion of the ~130 bp fragment in the double-variant nucleosome slowed down even though it continued to be degraded into smaller products in the H2A.Z nucleosome (middle graph in Figure 2C). This difference between the double-variant nucleosome and the H2A.Z nucleosome is small but statistically significant (Figure 2D). This observation suggests that the co-existence of H3.3 with H2A.Z on nucleosomes not only enhances terminal DNA accessibility but also provides DNA protection at internal sites within the nucleosome.

To corroborate the MNase results, we performed an additional assay employing the restriction enzyme HinfI, which targets a cleavage site proximal to SHL-6.5/6.5. The results show that HinfI digestion progresses notably faster for nucleosomes containing H2A.Z compared to canonical or H3.3 nucleosomes (Figure S1). Furthermore, it shows no significant difference in HinfI site accessibility between the H2A.Z nucleosome and the H2A.Z-H3.3 double-variant nucleosome. The outcome of this assay is consistent with the digestion pattern observed in the MNase assay concerning the 145 bp fragment (Figure 2B,C). Hence, our findings from the HinfI digestion assay reinforce those obtained from the MNase assay.

### 3.2. Cryo-EM Structure of the H2A.Z-H3.3 Double-Variant Nucleosome

**Figure 3:**
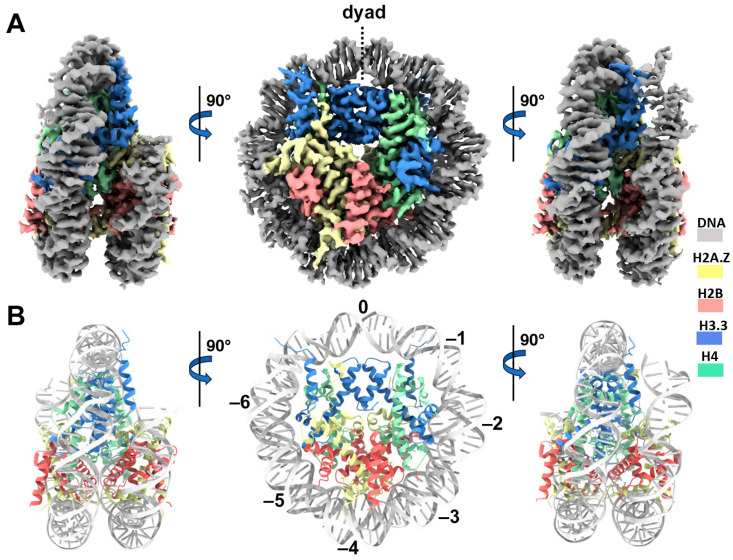
Cryo-EM structure of the H2A.Z-H3.3 double-variant nucleosome. (**A**) Surface representation of the cryo-EM density map of the double-variant nucleosome, shown in three different views. Densities of the DNA and different histones are color-coded according to the label shown on the right. (**B**) Atomic model of the double-variant nucleosome, displayed in three different views corresponding to those in (**A**). The color codes for different molecules correspond to those in (**A**). The superhelical locations are labeled with numbers for reference.

**Figure 4:**
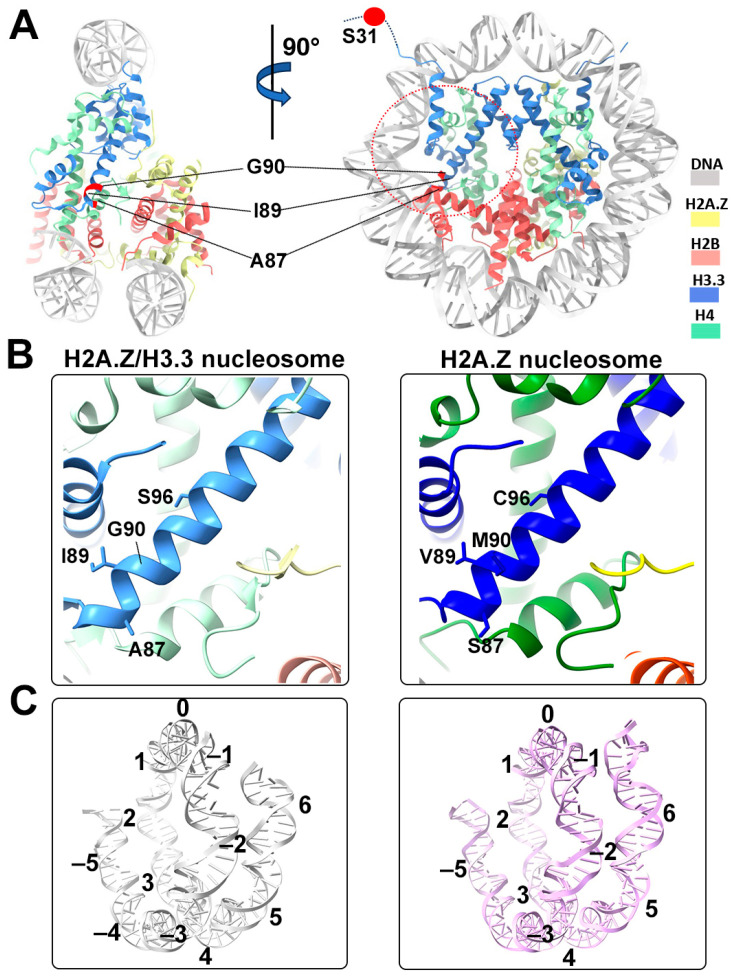
Structural comparison of the H2A.Z nucleosome and double-variant nucleosome. (**A**) The five H3.3-specific residues are highlighted and labeled in the current model. To enhance clarity and illustrate the structure surrounding H3.3, we utilized global clipping planes in ChimeraX to cut away part of the structure. The far clipping plane was employed to create the model’s side view on the left, while the near clipping plane was utilized to generate the view on the right. Densities of the DNA and different histones are color-coded according to the label shown on the right. (**B**) Close-up view of the H3.3 α2 helix region (red dotted oval in (**A**)) in the H2A.Z-H3.3 nucleosome (left). The same region of histone H3.1 in the H2A.Z nucleosome is shown (right, PDB ID 71MX). The four H3.3-specific residues and their counterparts in H3.1 are labeled. The near clipping plane was employed. (**C**) Comparison of the DNA of the H2A.Z-H3.3 nucleosome (left) with the DNA of the H2A.Z nucleosome, showing slightly longer/more resolved DNA ends beyond SHL6 in the H2A.Z nucleosome. The superhelical locations are labeled.

We next performed single-particle cryo-EM on the H2A.Z-H3.3 double-variant nucleosome. 3D classification of the dataset shows all classes with asymmetric DNA ends (Figure S2C). The final density map at a 3 Å resolution was calculated from the best class without imposing any symmetry (Figure 3A and Figure S2). Using this density map, we generated an atomic model of the double-variant nucleosome. The model closely resembles our previous H2A.Z nucleosome structure [23], featuring DNA asymmetrically wrapped around the histone core (Figure 4C). This asymmetric wrapping of the DNA is likely attributed to the asymmetry of the Widom 601 nucleosome-positioning sequence, as observed in other variant nucleosomes derived using the same synthetic DNA [33]. Previous studies indicate that variant H2A.Z accentuates this asymmetry compared to the canonical nucleosome [23].

One of the five H3.3-specific residues is located at the N-terminal tail of the protein, remaining disordered and thus unresolved in our structure (Figure 4A). The other four amino acid substitutions in variant H3.3 (A87, I89, G90, and S96) are part of the α2 helix, which adopts the same configuration as that in the canonical histone H3.1 from our previous H2A.Z nucleosome model (Figure 4B). These amino acids, like their counterparts in histone H3.1 (S87, V89, M90, C96), are part of the hydrophobic residue group. This suggests that the amino acid substitutions in variant H3.3 are unlikely to alter the protein structure and its interactions with the hydrophobic core at H4 [42]. Consequently, this elucidates why variant H3.3 alone has a minimal effect on nucleosome stability, as it is unlikely to modify the histone–DNA interactions near SHL-2/2.

### 3.3. INO80-Mediated Nucleosome Sliding on H2A.Z- H3.3 Double-Variant Nucleosome

Next, we sought to investigate the impact of variant H3.3 on ATP-dependent chromatin remodeling, specifically focusing on its potential influence on nucleosome–remodeler interactions and the remodeling efficiency. Notably, a recent proteomics study revealed an association of INO80 with H3.3, but not with H3.1 [43]. To further investigate the functional consequence of H3.3 on INO80 activity, we conducted in vitro nucleosome-sliding assays using the recombinant yeast INO80 complex (also known as INO80-C). The INO80 complex, an evolutionary conserved chromatin remodeler, is known to participate in various DNA metabolic processes, including transcription, replication, and damage repairs [44]. Like other members of the chromatin remodeler superfamily, INO80 exhibits typical remodeling activities such as mobilizing nucleosomes locally and influencing nucleosome spacing in vivo [44,45]. These functions are attributed to the enzyme’s capacity to reposition nucleosomes along DNA in an ATP-dependent manner [46]. Notably, H2A.Z nucleosomes are recognized as better substrates for INO80 compared to canonical nucleosomes in a nucleosome-sliding assay [47].

We used end-positioned nucleosomes (0N80) to assess the DNA translocation activity of INO80-C across various nucleosome substrates. Specifically, we compared the INO80-C-mediated nucleosome sliding on the H3.3, H2A.Z, and H2A.Z-H3.3 double-variant nucleosomes with canonical nucleosomes serving as the control. Our findings reveal that both the H2A.Z and H2A.Z-H3.3 nucleosomes equally enhance the DNA-translocation activity of the complex, evidenced by the fact that nearly 100% of the end-positioned nucleosomes were shifted to the center position after 10 min (Figure S3). In contrast, INO80 shifted only half of the canonical and H3.3 nucleosome substrates from the end to the center position (Figure S3). Consistent results were obtained from two additional independent experiments. These results suggest that variant H3.3 does not alter nucleosome properties that influence INO80-dependent nucleosome sliding.

## 4. Discussion

In our current study, we utilized Cryo-EM and biochemical assays to characterize the H2A.Z-H3.3 double-variant nucleosome. Our findings demonstrate that variant H3.3 incorporation does not significantly alter entry/exit DNA dynamics in nucleosomes. However, when variant H3.3 coexists with H2A.Z in the same nucleosome, the nucleosome exhibits enhanced terminal DNA mobility and accessibility at a level that is comparable to the H2A.Z nucleosomes. Our Cryo-EM analysis further supports this observation, revealing that H2A.Z-H3.3 double-variant nucleosomes adopt a nearly identical conformation to H2A.Z nucleosomes. The atomic model of the double-variant nucleosome shows that the four H3.3-specific residues in the ⍺2 helix do not cause detectable changes in any secondary structural elements in the histone core.

Overall, our results align with previous observations stating that H3.3 alone has minimal effects on nucleosome stability [31,32]. Notably, our MNase digestion assay reveals a small but significant difference between H2A.Z and double-variant nucleosomes, where DNA located at the internal sites shows greater protection in double-variant nucleosomes compared to in H2A.Z nucleosomes. This novel discovery suggests that the simultaneous presence of H3.3 and H2A.Z on the nucleosome introduces an additional mechanism to fine-tune DNA accessibility and thus the chromatin environment.

Finally, we demonstrate that the sole presence of variant H3.3 does not affect ATP-dependent INO80-mediated nucleosome sliding, while double-variant nucleosomes behave similarly to H2A.Z nucleosomes in stimulating the DNA translocation activity of INO80. These findings imply that H3.3-mediated changes on mono-nucleosomes primarily hinge on its co-occupancy with H2A.Z. However, the exact molecular mechanisms underlying the functional roles of variant H3.3 in transcription remain elusive. We speculate that a significant aspect of this mechanism involves the ability of H3.3 to recruit chromatin-associated proteins, along with its coordinated action with other histone variants to modulate higher-order chromatin structures. Future studies will delve into investigating how the H3.3 variant influences higher-order chromatin structures.

## Data Availability

The data that support the findings of this study are openly available in the Electron Microscopy Database (https://www.ebi.ac.uk/pdbe/emdb, accessed on 7 May 2024) and the Protein Data Bank (https://www.rcsb.org/, accessed on 7 May 2024). The EM map of the H2A.Z-H3.3 double-variant nucleosome is deposited in the Electron Microscopy Database under accession code EMD-44148. The corresponding protein coordinate is deposited in the Protein Data Bank under accession code PDB ID 9B3P.

## Supplementary Materials

The following supporting information can be downloaded at https://www.mdpi.com/article/10.3390/epigenomes8020021/s1: Figure S1: Assessment of nucleosome DNA accessibility by HinfI endonuclease cleavage; Figure S2: Single-particle cryo-EM data processing workflow; Figure S3: Effect of histone variants H3.3 and H2A.Z on Ino80-mediated nucleosome sliding; Table S1: Summary of cryo-EM data collection and model refinement.
