# Supplementary material for: Structural and Biochemical Characterization of the Nucleosome Containing Variants H3.3 and H2A.Z
Source: Epigenomes. 2024 May 27;8(2):21. doi: 10.3390/epigenomes8020021 (PMC11203148; doi:10.3390/epigenomes8020021)
Supplement: Supplementary file 1 [file epigenomes-08-00021-s001.zip › Sokolova_coVariant-Suppl_v1_proof.pdf]

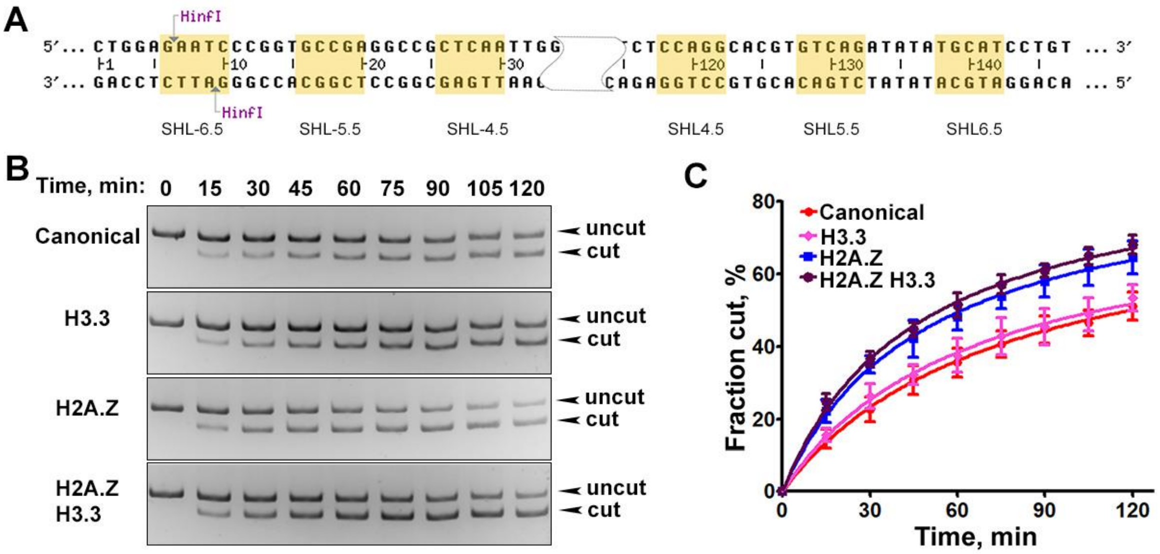

Figure S1. Assessment of nucleosome DNA accessibility by HinfI endonuclease cleavage.

(A) Illustration depicting the location of the HinfI cleavage site on the 601 Widom sequence used in this study, along with the corresponding SHL positions.

(B) Representative acrylamide gel showing the HinfI assay results of canonical, H3.3, H2A.Z and H2A.Z-H3.3 double-variant nucleosomes, respectively. The digestion product and initial DNA are labeled.

(C) Quantitative analysis of the HinfI digestion, as shown in (B). The graph presents the fraction of digested nucleosomes over time. Data points represent the average of three experiments. Data are mean ± SD.

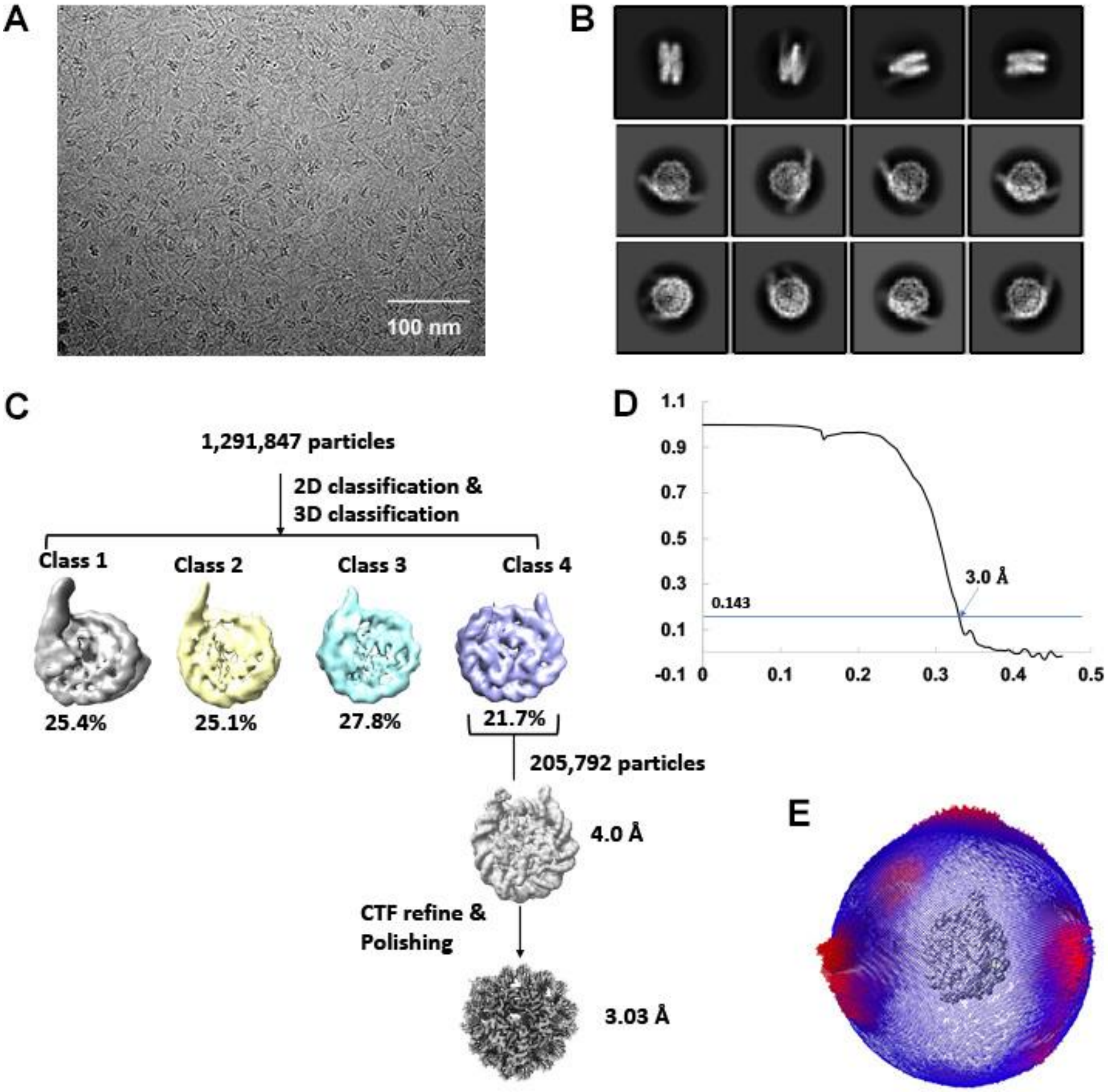

Figure S2. Single-particle cryo-EM data processing workflow.

- **(A)** Representative micrograph displaying the double-variant nucleosome embedded in vitreous ice. Scale bar is provided.
- **(B)** Selected 2D class averages of the double-variant nucleosome in different views.
- **(C)** Sequential steps involved in the single-particle data analysis of the cryo-EM dataset.
- **(D)** Gold-standard Fourier Shell Correlation (FSC) curve showing the final consensus refined map (with masking) at an average resolution of 3Å.
- **(E)** Angular distribution plot depicting the orientation of particles used for generating the final consensus refined map.

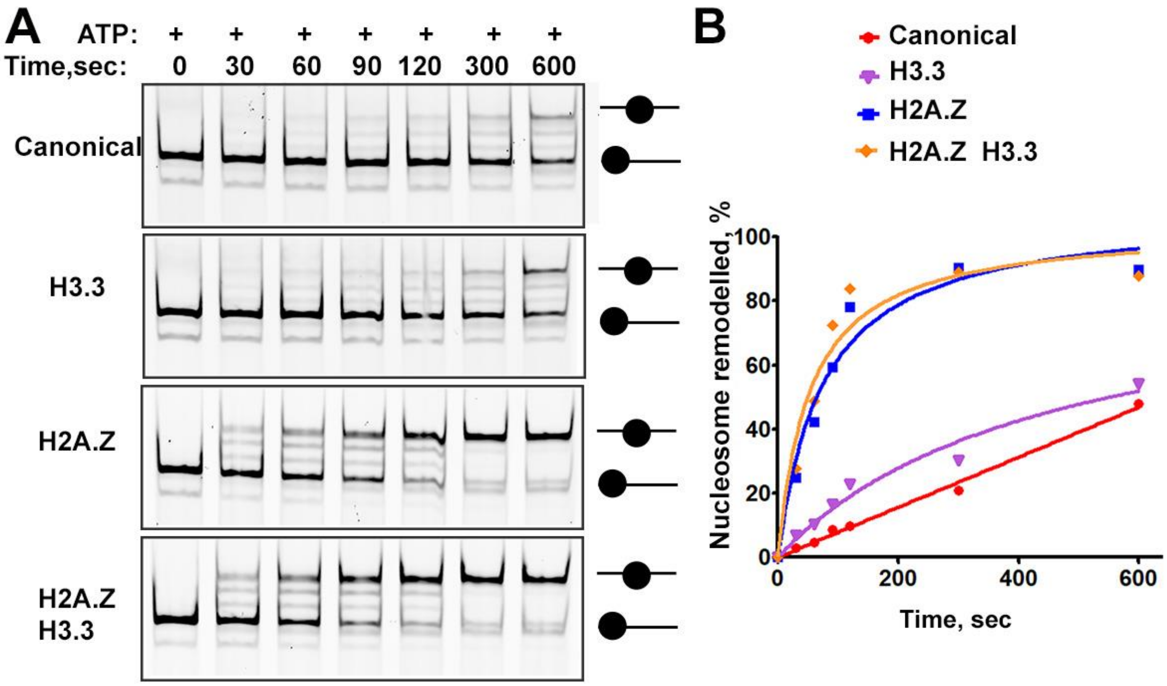

Figure S3. Effect of histone variants H3.3 and H2A.Z on INO80-mediated nucleosome sliding.

- **(A)** The outcomes of INO80-C-mediated nucleosome-sliding on canonical, H3.3, H2A.Z and H2A.Z-H3.3 nucleosomes, respectively, revealed by Native-PAGE. Schematic representations on the right side of the gels denote the end-positioned and center-positioned nucleosome bands.
- **(B)** Quantitative analysis of the results presented in (A). The graph illustrates the fraction of remodeled nucleosomes over time.

Supplemental Table S1 | Summary of cryo-EM data collection and model refinement

Cryo-EM data collection, refinement and validation statistics

	EMDB-44148
**Data collection and processing**	
Magnification	81,000
Voltage (kV)	300
Total electron exposure (e-/Å^2)	50
Defocus range (μm)	1.0-2.25
Pixel size (Å)	1.08
Symmetry	C1
Initial particle images (no.)	1,291,847
Final particle images (no.)	205,792
Map resolution (Å)	3.0
FSC threshold	0.143
Sphericity of 3DFSC	0.9667
Map sharpening *B* factor (Å)	87.4
**Model refinement and validation**	
Initial model used (PDB code)	5B33, 6FQ5
Model resolution (Å)	3.0
Model-to-map fit, CC_mask	0.8161
Validation	
MolProbity score	1.75
Clashscore	6.00
Rotamer outlier (%)	1.97
Ramachandran plot	
Favored (%)	96.81
Allowed (%)	3.19
Outlier (%)	0.00
